# Successful Removal of the Uterus and Both Ovaries, by Abdominal Section, the Tumor Fibro-Cystic, Weighing Thirty-Seven Pounds

**Published:** 1866-04

**Authors:** Horatio Robinson Storer

**Affiliations:** Boston, Assistant in Obstetrics, &c., &c.


"*Successful Removal of the Uterus and both Ovaries, by abdominal section, the Tumor Fibro-cystic, weighing thirty-seven pounds.*—By Horatio Robinson Storer, M. D., of Boston, Assistant in Obstetrics," &c., &c.

We are in receipt of a phamphlet of 32 pages, bearing the above title. From the statistical researches of Dr. Storer it appears that his is the sixth successful extirpation of the uterus by abdominal section, upon record. Out of twenty-four operations of the kind published, eighteen proved fatal, leaving the "per centage of recoveries 1 in 4, or 25 per cent."

Though, at first view, the work savors somewhat of egotism, yet we freely and willingly pass by the slight foibles of eminent brethren in the profession whose lives are spent in ameliorating the condition of suffering humanity.

The operation detailed in the work is one of importance, and he who relieves the poor sufferer from the burthen life carries with it, by such timely successful operation, deserves to be called a benefactor and philanthropist.

